# Targeting Lactate and Lactylation in Cancer Metabolism and Immunotherapy

**DOI:** 10.1002/advs.202515403

**Published:** 2026-01-12

**Authors:** Jiajing Gong, Yuhang Xie, Zhijie Weng, Yongzhi Wu, Longjiang Li, Bo Li

**Affiliations:** ^1^ State Key Laboratory of Oral Diseases, National Center for Stomatology, National Clinical Research Center for Oral Diseases, Department of Head and Neck Oncology, West China Hospital of Stomatology Sichuan University Chengdu Sichuan China; ^2^ State Key Laboratory of Oral Diseases, National Center for Stomatology, National Clinical Research Center for Oral Diseases, Department of Orthodontics, West China Hospital of Stomatology Sichuan University Chengdu Sichuan China

**Keywords:** cancer, lactate, lactylation, metabolism, tumor microenvironment (TME)

## Abstract

Lactate has evolved from being regarded as a byproduct of glycolysis to a pivotal regulator of cancer metabolism and signaling. The Warburg effect underscores how elevated lactate production meets the biosynthetic demands of highly proliferative cancer cells, while shaping an immunosuppressive tumor microenvironment (TME) that supports cancer growth and metastasis. The discovery of lactylation, a novel post‐translational modification, has further expanded the conceptual landscape, revealing how lactate serves as both a metabolite and a signaling molecule that couples metabolic reprogramming with gene regulation. This review delineates how lactate dynamically shuttles through the TME and boosts cancer malignancy, including proliferation, metastasis, drug resistance, and immune evasion. Also, this review integrates and discusses how lactate‐driven lactylation bridges metabolic and epigenetic control. Furthermore, emerging therapeutic strategies targeting lactate metabolism and lactylation are summarized, revealing their promise in cancer immunotherapy. Collectively, a comprehensive perspective is provided on the multifaceted roles of lactate and lactylation in cancer biology and, more importantly, highlights potential translational avenues for clinical applications.

## Graphical abstract

Lactate, once deemed a metabolic waste, emerges as a central regulator of cancer progression. This review elucidates how lactate and its epigenetic derivative, protein lactylation, orchestrate tumor metabolism, immune suppression, and therapeutic resistance. It further highlights evolving strategies that target lactate metabolism and lactylation to enhance cancer immunotherapy and clinical translation.

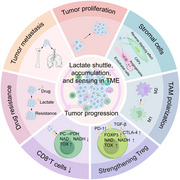

## 1 Introduction

During the multistep development of human cancers, cancer cells acquire a set of biological signatures that together drive malignant progression [1]. Among these, metabolic reprogramming has emerged as a core hallmark, sustaining uncontrolled proliferation and conferring resistance to cell death [1, 2]. In the 1920s, Otto Warburg observed that cancer cells consume more glucose and preferentially rely on aerobic glycolysis even in normoxic conditions [2]. This metabolic shift results in marked lactate accumulation inside cancer cells and in their surrounding environment, spurring growing interest in its biological significance. As such, once dismissed as a waste product under hypoxic conditions, lactate is now recognized as a versatile metabolite with profound effects on cancer progression and clinical outcomes [3, 4, 5].

Lactate accumulation also reflects a fundamental shift in cellular metabolic state. Intracellular lactate rises sharply when glycolytic flux exceeds mitochondrial oxidative capacity, enabling lactate to function as a sensor and readout of cellular metabolism [6]. Elevated lactate marks the transition from oxidative phosphorylation (OXPHOS) to glycolysis [6], contributing to mitochondrial redox pressure, stabilizing hypoxia‐inducible factor (HIF) activity, and reinforcing glycolytic gene expression [7, 8]. Thus, lactate accumulation forms a feed‐forward loop that commits cancer cells to glycolysis, characteristic of the Warburg phenotype, effectively coupling metabolic stress to transcriptional responses.

The lactate shuttle occurs across a wide range of physiological and pathological events [9]. Lactate and protons move down their concentration gradients, enabling dynamic exchange within and between cells [10]. Although lactate cannot freely cross the plasma membrane in anionic form, its transport depends on monocarboxylate transporters (MCTs) [11]. MCTs are encoded by members of the solute carrier family 16 (SLC16), which comprises 14 transporters responsible for shuttling monocarboxylates, protons, and related metabolites. Among them, it is MCT1–MCT4 that mediate bidirectional, proton‐coupled lactate transport [11], allowing lactate to serve not only as an energy substrate and gluconeogenic precursor but also as a signaling messenger that coordinates metabolic activities across the tumor microenvironment (TME) [10].

Within the TME, cancer cells exhibit significant metabolic heterogeneity, shaped by their spatial proximity to blood vessels [12]. Accordingly, malignant cells can be categorized into normoxic or hypoxic, each adopting distinct metabolic programs [12]. Hypoxic cancer cells convert pyruvate to lactate via lactate dehydrogenase A (LDHA), releasing lactate that can be taken up and oxidized by neighboring normoxic cancer cells. This metabolic symbiosis also occurs between cancer cells and stromal cells [13]. Cancer‐associated fibroblasts (CAFs), for example, upregulate glucose transporters type 1 (GLUT1) and MCT4 to increase glucose uptake and lactate export [14]. In contrast, cancer cells downregulate GLUT1 expression and increase MCT1 expression to import CAF‐derived lactate for OXPHOS—a phenomenon known as the reverse Warburg effect [14]. These adaptive exchanges underscore lactate's central role in supporting tumor resilience and metabolic flexibility.

Limited vascularization and rapid cell proliferation in solid tumors further constrain oxygen and glucose availability, leading cancer cells to exploit alternative energy sources [15]. Stable isotope tracing with ^13^C‐labeled metabolites demonstrates that cancer cells actively take up and oxidize lactate in vivo [16, 17, 18]. Under glucose‐limited conditions, lactate and glutamine contribute to nicotinamide adenine dinucleotide phosphate (NADPH) generation via isocitrate dehydrogenase 1 (IDH1) and malate dehydrogenase 1 (MDH1), respectively [19]. Lactate serves as a major carbon source for the tricarboxylic acid (TCA) cycle in diverse cancers [6, 16, 17], especially in lung cancers [17]. Enhanced lactate metabolism also correlates with metastatic potential; in melanoma, highly metastatic cells display increased lactate uptake driven by upregulated MCT1 activity [20]. These observations reinforce lactate as a critical metabolic fuel and highlight the therapeutic promise of targeting lactate in cancer.

Beyond its metabolic functions, lactate also contributes to epigenetic modification [21, 22]. In 2019, lysine lactylation (Kla) was identified on histone proteins, derived from intracellular lactate [21]. This post‐translational modification (PTM) regulates gene expression and influences diverse cellular processes, including proliferation, differentiation, and immune responses. Emerging evidence indicates that lactylation is widespread across the human proteome, particularly enriched in glycolytic enzymes [23]. In cancer, enhanced histone lactylation promotes oncogene expression; studies in colorectal and breast cancer have demonstrated that increased histone lactylation correlates with poor prognosis in patients [24, 25]. These recent findings reveal a new dimension of lactate biology—metabolic activity can directly shape chromatin states and cellular phenotypes.

In this review, we synthesize current knowledge on the multifaceted roles of lactate within the TME, highlighting how lactate and lactylation interconnect metabolic reprogramming with epigenetic control in cancer progression. We also summarize emerging therapeutic strategies targeting lactate metabolism and histone lactylation, and discuss their potential to counteract tumor‐associated immunosuppression and enhance the efficacy of cancer immunotherapy.

## 2 Lactate as a Metabolite in the Tumor Microenvironment

**FIGURE 1:**
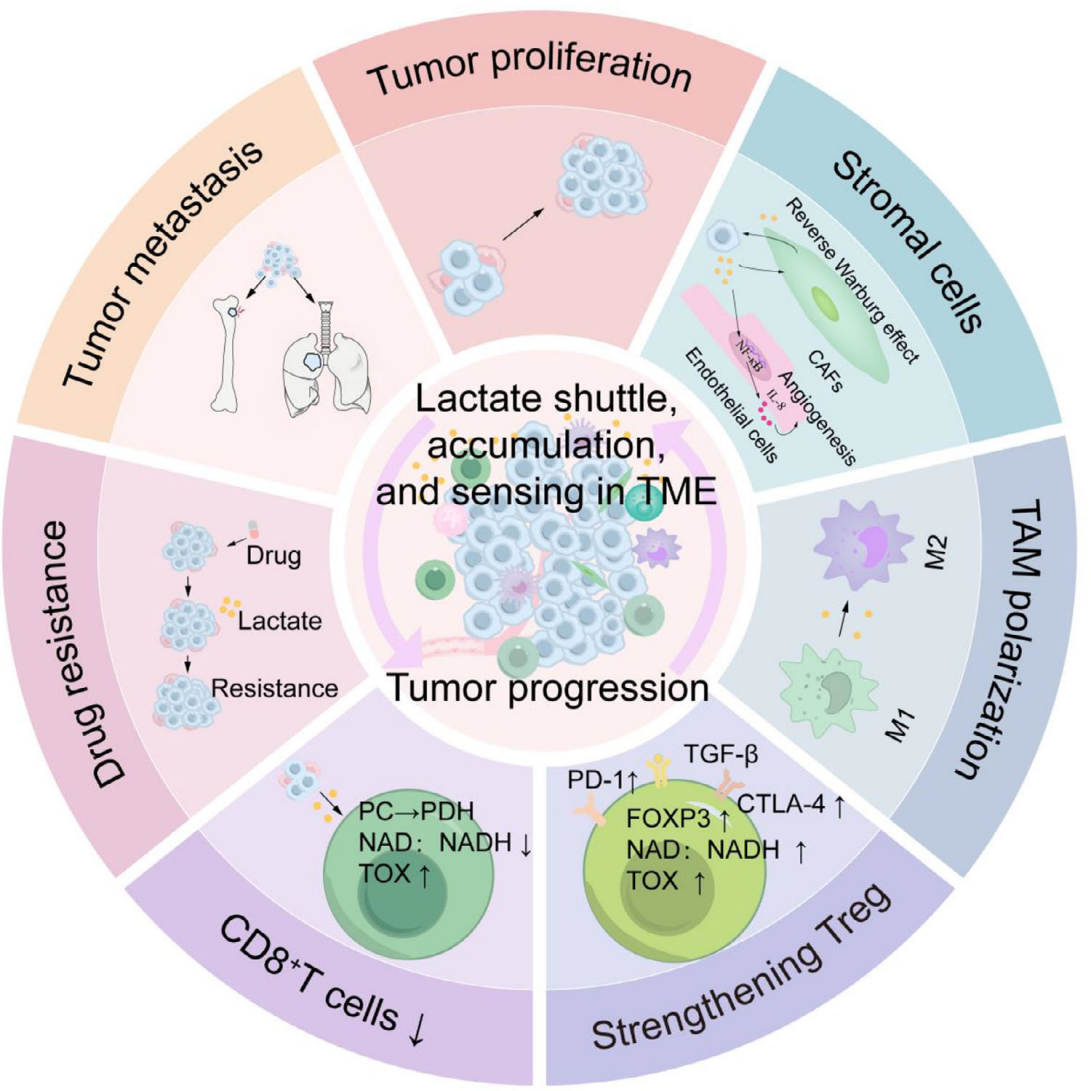
**The role of lactate in the tumor microenvironment (TME)**. Lactate promotes tumor proliferation, metastasis, and drug resistance. Lactate reshapes the tumor immune suppressive microenvironment, which involves the inhibition of CD8^+^ T‐cell activity, the promotion of Treg infiltration and immune suppressive effects, and the promotion of TAM polarization toward the M2 pro‐tumor phenotype. Lactate also causes stromal cells in the TME to undergo pro‐tumor reprogramming. Abbreviations: CD8^+^ T, Cytotoxic T lymphocyte; CTLA‐4, Cytotoxic T‐lymphocyte‐associated protein 4; FOXP3, Forkhead box P3; NAD, Nicotinamide adenine dinucleotide (oxidized form); NADH, Nicotinamide adenine dinucleotide (reduced form); PC, Pyruvate carboxylase; PD‐1, Programmed cell death protein 1; PDH, Pyruvate dehydrogenase; TAM, Tumor‐associated macrophage; TGF‐β, Transforming growth factor beta; TME, Tumor microenvironment; TOX, Thymocyte selection‐associated high mobility group box protein.

TME represents a highly organized and dynamic ecosystem comprising heterogeneous cancer cells, resident and/or recruited stromal compartments—including immune cells, CAFs, endothelial cells, and pericytes—embedded within a vascularized extracellular matrix (ECM) [26]. Elevated lactate levels in the TME acidify the local milieu, creating conditions that favor cancer progression [27]. Increasing evidence indicates that lactate exerts regulatory effects on both cancer cells and various non‐malignant cells within this niche (Figure 1).

### 2.1 Effects of Lactate on Cancer Cells

Lactate has become recognized as a central regulator of cancer cell behavior, extending beyond its traditional metabolic role. Within tumor cells, lactate shapes metabolic networks and signaling pathways that together influence growth, survival, and adaptability. Elevated lactate levels have been shown to meet biosynthetic demands, sustain proliferation, enhance metastatic potential, and promote resistance to therapy through diverse molecular mechanisms.

#### 2.1.1 Promotion of Cancer Progression

Lactate facilitates cancer progression through multiple mechanisms [12, 28, 29]. It can directly modulate the cell cycle and promote cell proliferation [30] and, through systemic effects, promote the release of small extracellular vesicles (sEVs) derived from cancer cells that facilitate intercellular communication. Elevated plasma lactate levels in cancer patients correlate with increased sEVs production, underscoring its role as both a metabolic and signaling mediator [31]. In parallel, senescent cells undergo metabolic alterations that increase lactate output, further fueling malignant progression within the TME [32].

A key molecular conduit for these effects is G protein‐coupled receptor 81 (GPR81), which is highly expressed in various cancer types and crucial for cancer cell survival. Lactate activates signal transducer and activator of transcription 3 (STAT3), which binds to the GPR81 promoter to upregulate its transcription [33], and concurrently acts in an autocrine fashion to stimulate surface GPR81 signaling [34]. Activated GPR81 coordinates the expression of genes required for lactate transport and metabolism; its silencing suppresses peroxisome proliferator‐activated receptor gamma coactivator 1‐alpha (PGC‐1α), MCT1, MCT4, and their chaperone protein CD147, resulting in diminished mitochondrial activity [35, 36, 37]. Although the signaling network remains unresolved, GPR81 is found to regulate the NOTCH ligand delta‐like 4 (DLL4) in the TME, potentially modulating genes associated with ECM remodeling, cell adhesion, and signal transduction [38]. Moreover, recent evidence also demonstrates lactate‐induced GPR81 activation in the pathogenesis of cancer cachexia [39].

In addition, lactate contributes to the maintenance of cancer stem cell (CSC) phenotypes. CSCs depend on flexible metabolic programs to self‐renew and resist differentiation, and lactate functions as a crucial cue within the TME. Recent studies demonstrate that lactate sustains CSC stemness by activating WNT signaling and reinforcing the metabolic coupling between CSCs and surrounding non‐CSCs through MCT‐dependent lactate exchange in organoid models [40, 41, 42]. This lactate‐mediated plasticity enhances cancer cell adaptability and therapeutic resistance, but it also exposes a metabolic vulnerability: inhibiting lactate utilization may deplete the CSC pool and impair tumor maintenance.

#### 2.1.2 Enhancement of Cancer Metastasis

As mentioned above, elevated lactate secretion acidifies the TME, and increasing evidence links this acidic milieu to metastatic progression [43, 44, 45]. Lactate facilitates cytoskeletal remodeling and enhances cell adhesion and migration, thereby promoting local invasion and dissemination [46, 47]. Acidification also triggers a cascade of pro‐metastatic factors, including vascular endothelial growth factor (VEGF), matrix metalloproteinases (MMPs), cathepsins, and transforming growth factor‐β (TGF‐β), which collectively drive angiogenesis, ECM degradation, and epithelial–mesenchymal transition [45]. In hypoxic tumors, lactic acidosis induces TGF‐β expression, which in turn stimulates fatty acid synthesis and upregulates VEGF and MMPs, promoting angiogenesis and invasion [45]. Consistently, cancers with high lactate uptake display increased metastatic potential [20]. However, blood vessels and oxygen are unevenly distributed in solid tumors. Hypoxic cells, despite their stronger metastatic traits, are less likely to intravasate because of their greater distance from blood vessels [44]. Instead, these cells release lactate into the TME, which fuels nearby normoxic CSCs via PGC‐1α‐mediated OXPHOS, enhancing their metastasis [44].

Lactate also plays a critical role in shaping the metastatic niche, particularly in bone metastasis [48]. By activating cAMP‐response element binding protein (CREB) and mechanistic target of rapamycin (mTOR) signaling, lactate induces CXCL10 and cadherin‐11 (CDH11) in CD115^+^ osteoclast precursors, thereby promoting their differentiation, adhesion, and functional maturation to establish a bone microenvironment permissive for cancer cell colonization [48]. In mouse xenograft models, lactate exported through MCT4 further remodels the bone metastatic niche by reinforcing tumor‐stromal interactions and supporting invasive growth within the skeletal compartment [49, 50]. Collectively, these findings highlight lactate as a key metabolic cue that orchestrates cytoskeletal remodeling, proteolytic activity, and niche conditioning during bone metastasis, suggesting that targeting lactate‐driven signaling may limit metastatic progression and improve patient prognosis and quality of life.

#### 2.1.3 Induction of Therapeutic Resistance

Metabolic rewiring is a central driver of drug resistance in cancer [51, 52, 53]. Prolonged treatment with tyrosine kinase inhibitors induces adaptive metabolic shifts, marked by increased glycolysis and lactate output [54]. Within the TME, lactate secreted by cancer cells mediates crosstalk between cancer cells with tumor‐associated macrophages (TAMs) [55] and CAFs [56], fostering a pro‐tumorigenic niche that supports drug tolerance. In estrogen receptor (ER)‐positive breast cancer, tamoxifen‐resistant cancer cells exhibit elevated glycolytic activity and protective autophagy, processes closely associated with lactate metabolism through key regulators, such as MCT1, lactate dehydrogenase A (LDHA), and LDHB, which mediate lactate influx and transformation under therapeutic stress [57, 58]. Likewise, resistance to phosphoinositide 3‐kinase (PI3K)/mTOR inhibitors is exacerbated when breast cancer cells exploit lactate as an alternative oxidative fuel [59]. Beyond its metabolic roles, lactate confers chemoresistance by enhancing DNA repair capacity via regulation of ATP‐binding cassette (ABC) transporters, which facilitate drug efflux and limit cytotoxic efficacy [60]. This mechanism underlies resistance to multiple chemotherapeutic agents, such as doxorubicin [61] and etoposide [62]. These findings underscore the potential of targeting lactate metabolism to overcome drug resistance.

### 2.2 Effects of Lactate on Immune Cells

Within the TME, lactate accumulates to levels far exceeding those found in normal tissues. Physiological lactate concentrations in blood and healthy tissues typically range from 1.5 to 3 × 10^−3^ m, whereas in cancer tissues they frequently reach 10–30 × 10^−3^ m, with some highly glycolytic tumor regions reported to contain levels approaching 40 × 10^−3^ m. This substantial buildup of lactate profoundly shapes immune cell behavior. Elevated lactate dampens T‐cell effector function, promotes the recruitment, differentiation, and activity of regulatory T cells (Tregs), and drives macrophage polarization toward a pro‐tumorigenic M2 phenotype, collectively contributing to an immunosuppressive milieu that supports cancer progression and facilitates immune evasion.

#### 2.2.1 Impact on CD8^+^ T Cells

Cancer‐derived lactate drives the metabolic rewiring of CD8^+^ cytotoxic T lymphocytes (CTLs), leading to functional decline [63]. These effector T cells rely on pyruvate carboxylase (PC) to maintain mitochondrial metabolism and supply intermediates of the TCA cycle [63]. At the metabolic branch point between PC and pyruvate dehydrogenase (PDH), lactate redirects pyruvate flux toward PDH‐mediated oxidation, thereby reducing the replenishment of TCA‐cycle metabolites and weakening CTL effector activity. Notably, pharmacological inhibition of PDH can restore CTL function [63].

In parallel, NAD^+^ biosynthesis represents a key checkpoint for T‐cell receptor (TCR)‐dependent activation and early metabolic programming [64]. l‐Lactate disrupts the NAD^+^: NADH redox balance, impairing glycolytic flux at the NAD^+^‐dependent enzyme glyceraldehyde‐3‐phosphate dehydrogenase (GAPDH). This blockade reduces post‐GAPDH glycolytic intermediates and serine availability, both of which are essential for CTL proliferation and function [65]. Furthermore, cancer‐driven metabolic remodeling within the TME compromises naïve T cell survival by disturbing the transcriptional balance between pro‐apoptotic and anti‐apoptotic gene expression [66].

Intratumoral CD8^+^ T cells commonly acquire an exhausted phenotype [26]. The transcription factor TOX is highly expressed in dysfunctional, tumor‐specific T cells [67]. In acute myeloid leukemia (AML), elevated lactate levels induce TOX expression, leading to the functional exhaustion of CD8^+^ T cells and suppressing the expression of key cytolytic effectors, including perforin and granzyme B (GZMB) [68].

#### 2.2.2 Impact on Tregs

Tregs are considered a major barrier to anti‐tumor immunity due to their immunosuppressive potency. They accumulate within the TME and undergo profound metabolic adaptation to thrive under nutrient‐deprived, lactate‐rich conditions [69]. Lactate reinforces Treg function by upregulating FOXP3, the master transcription factor of Tregs. Unlike effector T cells, whose metabolism is suppressed by lactate‐induced NAD^+^ depletion via LDH, Tregs maintain metabolic fitness through FOXP3‐dependent rewiring, which increases the NAD^+^: NADH ratio and confers a survival advantage in low‐glucose, high‐lactate environments [70].

CAFs further potentiate Treg expansion by upregulating NF‐κB‐driven FOXP3 expression in response to lactate signaling [71]. Meanwhile, lactate‐GPR81 signaling facilitates Treg infiltration by increasing CX3CL1 expression in the TME [72]. In the highly glycolytic TME, Tregs import lactate via MCT1, which promotes NFAT1 nuclear translocation and subsequently upregulates programmed cell death protein 1 (PD‐1) [73]. Similarly, in AML, elevated lactate drives the accumulation of PD‐1^+^ Tregs within the bone marrow niche [74]. Furthermore, lactate uptake facilitates the expression of CTL‐associated protein 4 (CTLA‐4), a key immune checkpoint receptor involved in immune tolerance and suppressing anti‐tumor responses [75].

#### 2.2.3 Impact on TAMs

TAMs exhibit substantial functional plasticity and phenotypic heterogeneity [76]. Macrophages are broadly categorized into two major subsets based on their surface markers and functions: pro‐inflammatory M1 macrophages, which mediate anti‐tumor responses, and anti‐inflammatory M2 macrophages, which support tissue repair and are generally associated with tumor progression [77]. Within the TME, lactate plays a central role in driving TAM polarization toward the M2 phenotype [76, 77].

Lactate promotes M2‐like characteristics by upregulating interleukin‐6 (IL‐6), IL‐10, and HIF1α, while suppressing NF‐κB activity [78]. In microglia, lactate exposure decreases inducible nitric oxide synthase (iNOS), a canonical M1 marker, and increases arginase‐1 (ARG1), an M2 marker, consistent with an immunosuppressive phenotype [79]. Cancer‐derived lactate also induces HIF1α‐dependent macrophage polarization, which elevates the expression of ARG1 and VEGF, highlighting the lactate‐HIF1α axis as a critical signaling pathway that couples metabolic reprogramming with macrophage polarization [80]. Notably, though early pharmacological studies implicated mitochondrial pyruvate carrier (MPC)‐mediated metabolism in TAM polarization [81], subsequent genetic studies showed that MPC deletion did not affect HIF1α stabilization or histone lactylation, suggesting that lactate itself, rather than its downstream metabolites, is the principal driver of TAM polarization [82].

### 2.3 Effects of Lactate on Stromal Cells

Activated stromal cells promote tumor progression through reciprocal interactions. In the so‐called reverse Warburg effect, CAFs supply lactate to cancer cells, while cancer cell‐derived lactate reinforces the pro‐tumorigenic phenotype of CAFs by downregulating p62 [83]. Beyond this coupling, researchers demonstrate that CAFs undergo extensive lactate‐driven metabolic rewiring, which enhances their bioenergetic capacity and sustains their activated state [14]. Lactate also drives extensive epigenomic reprogramming during CAF differentiation, shaping their secretory profiles [84]. In particular, tumor‐derived lactate increases α‐ketoglutarate production in mesenchymal stem cells (MSCs), which activates the demethylase TET enzyme, leading to DNA demethylation‐driven epigenomic reprogramming that converts MSCs into pro‐invasive CAFs [84]. By fueling CAF metabolism, lactate supports their proliferation and enhances the secretion of IL‐6 and brain‐derived neurotrophic factor (BDNF), sustaining fibrosis, dampening anti‐tumor immunity, and promoting acquired drug resistance [56, 85]. Moreover, lactate‐activated CAFs increase ECM deposition and LOX‐mediated crosslinking, resulting in stromal stiffening that enhances mechanotransduction and facilitates cancer cell invasion.

In endothelial cells, lactate is imported via MCT1 and activates the autocrine NF‐κB/IL‐8 (CXCL8) signaling pathway, promoting angiogenesis [86]. Lactate also enhances endothelial metabolic fitness by promoting glycolytic flux and vessel sprouting, further supporting neovascularization in the TME [13]. Remarkably, crosstalk between tumors and the stromal compartments of cancer‐draining lymph nodes can occur even before overt metastasis. Structural and functional remodeling of these pre‐metastatic niches is driven, at least in part, by lactate‐induced reprogramming of fibroblastic reticular cells (FRCs) [87]. Cancer‐derived lactate transported to lymph nodes upregulates podoplanin (PDPN) and THY1, downregulates IL‐7, induces CAF‐like phenotypes, and impairs mitochondrial function in FRCs [87], thereby altering lymph node architecture, reducing immune‐cell trafficking, and priming the milieu for metastatic seeding [26], collectively creating a metastasis‐favorable TME.

## 3 Lactylation Reveals a New Dimension of Lactate Function

Epigenetic modifications regulate gene expression without altering DNA sequence and coordinate diverse downstream biological effects [88]. Lactylation has recently emerged as a widespread PTM affecting both histone and non‐histone proteins [23, 89]. The identification of a distinct signature fragment ion, cyclic ammonium ion, in tandem mass spectrometry spectra of lysine lactylation (Kla)‐containing peptides enabled reliable detection of this modification [23]. Since its discovery, researchers have found that lactylation plays a significant role in health and disease, including cancer. Current evidence suggests that lactylation serves as a key metabolic‐epigenetic interface that influences tumor initiation, stress adaptation, and therapy resistance.

### 3.1 Lactylation as a Novel Post‐Translational Modification

Structurally, Kla occurs in three isomer forms, including l‐lactyl‐lysine (Kl‐la), N‐ε‐(carboxyethyl)‐lysine (Kce), and d‐lactyl‐lysine (Kd‐la) [167, 168], among which Kl‐la predominates on histones and varies with glycolytic flux. Notably, Kl‐la is primarily derived from l‐lactate through the enzymatic generation of l‐lactyl‐CoA, whereas Kd‐la arises mainly through non‐enzymatic glycation driven by the methylglyoxal (MGO) pathway, which produces d‐lactyl‐glutathione and subsequently d‐lactyl‐CoA capable of modifying lysine residues through non‐enzymatic mechanisms. Unless otherwisespecified, all subsequent mentions of Kla refer specifically to the enzymatic Kl‐la modification.

**FIGURE 2:**
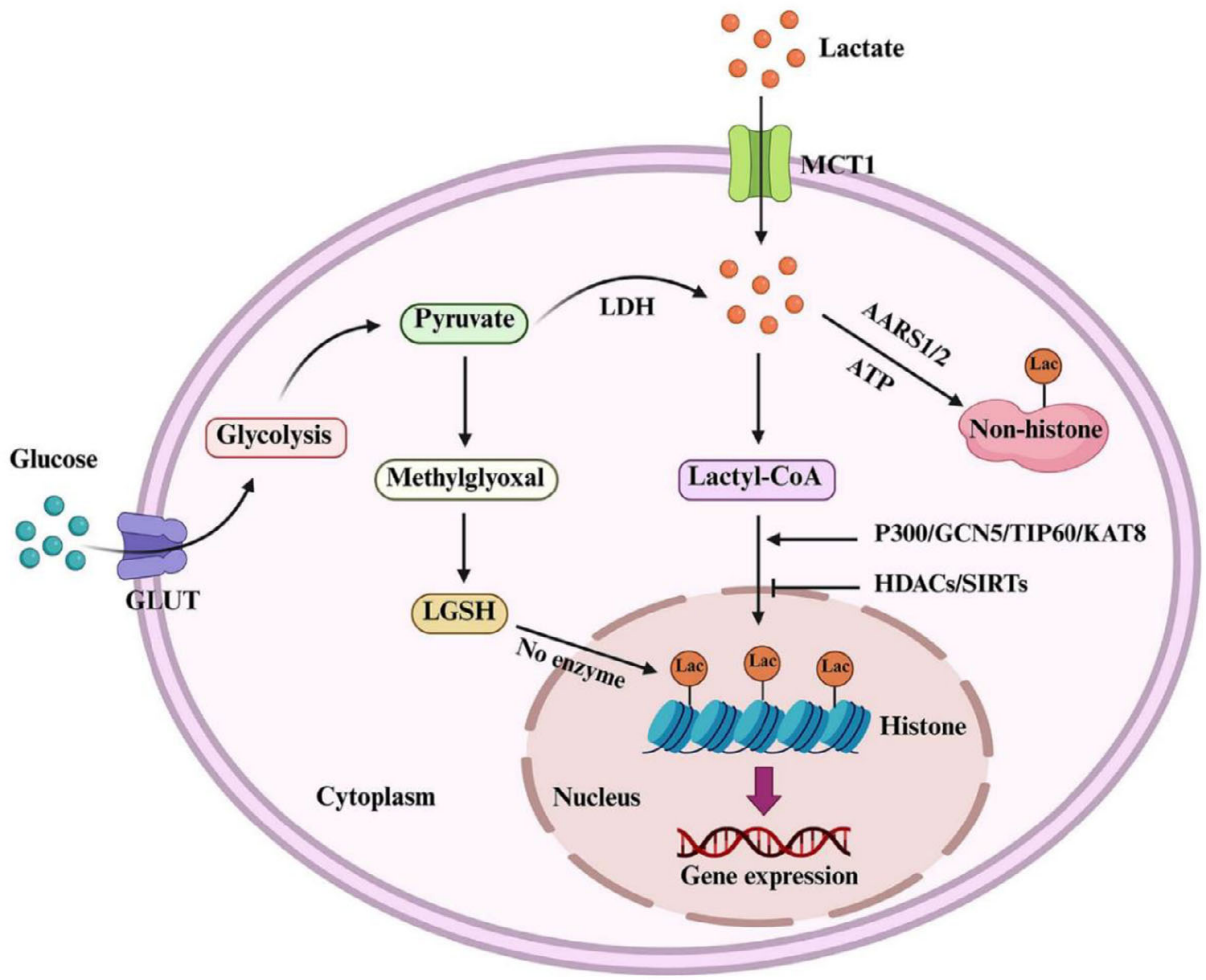
**Enzymatic regulation of histone lactylation**. Key enzymes involved in histone lactylation are being gradually identified, including lactyltransferases that catalyze the addition of lactyl groups and delactylases that remove them. Deciphering these regulatory enzymes provides mechanistic insight into the dynamic modulation of lactylation and its role in cancer epigenetics. Abbreviations: AARS, alanyl‑tRNA synthetase; GCN5, general control non‑depressible 5; GLUT, glucose transporter; HDAC, histone deacetylase; KAT8, lysine acetyltransferase 8; Lac, lactate; LDH, lactate dehydrogenase; LGSH, l‑lactyl coenzyme A (L‑La‑CoA) and S, d‑lactoylglutathione; MCT1, monocarboxylate transporter 1; SIRT, sirtuin; TIP60, tat‑interactive protein 60. Reproduced with permission [281]. 2025, Spandidos Publications.

Because Kl‐la is enzymatically generated, its regulation falls within the established paradigm of chromatin‐modifying enzymes that control other histone PTMs. In this canonical model, histone modifications are installed, interpreted, and removed by coordinated networks of “writers,” “readers,” and “erasers.” [88, 90] In this context, histone lactylation functions primarily as an epigenetic modification that links cellular metabolic state to transcriptional regulation. Kla conforms to this conceptual framework, and several core enzymes responsible for histone lactylation have now been identified (Figure 2) [90, 281].

A key step in Kla is the conversion of lactate into lactyl‐CoA, the acyl donor for lactylation, generated by acyl‐CoA: lactate CoA‐transferases (ALCTs) [90]. Protein lactylation occurs via two distinct routes: a canonical lactyl‐CoA‐dependent pathway and a recently identified lactyl‐CoA‐independent pathway [91]. Though both pathways contribute to histone and non‐histone protein lactylation, their relative importance and functional consequences depend on protein identity, subcellular localization, and tumor context. The formation of lactyl‐CoA alone is insufficient for lactylation; its utilization depends on the activity of dedicated acyltransferases that catalyze the transfer of the lactyl moiety to proteins. In mammals, acyltransferases are broadly categorized into three families: p300/CREB‐binding protein (CBP), the MYST (MOZ‐Ybf2/Sas3‐Sas2‐Tip60) family, including MOZ, KAT8, HBO1, and TIP60, and GCN5‐related N‐acetyltransferases (GNATs) family, including KAT2A, KAT2B, and HAT1 [91]. These enzymes share structurally similar catalytic pockets, enabling them not only to acetylate histone lysines but also to transfer alternative acyl groups, including lactyl groups, onto proteins [91].

In the canonical lactyl‐CoA‐dependent pathway, lactate is first converted into lactyl‐CoA, which then serves as an acyl donor for histone acyltransferases to modify lysine residues on proteins [91]. Two types of lactyl‐CoA synthetases have been identified: acetyl‐CoA synthetase 2 (ACSS2) [92] and guanosine triphosphate (GTP)‐specific succinyl‐CoA synthetase (GTPSCS) [93]. Recent studies have revealed that multiple acyltransferases contribute to histone and non‐histone lactylation in a tumor‐specific manner. For instance, p300 overexpression modestly increases global histone Kla levels, whereas its depletion causes a marked decline in lactylation [21]. In glioma, KAT2A acts as an active histone lactyltransferase, promoting cancer progression and immune evasion by enhancing lactylation‐driven transcriptional programs [92]. Moreover, KAT8‐mediated hyperlactylation of eukaryotic elongation factor 1A2 (EEF1A2) has been detected in human colorectal cancer, linking KAT8 to aberrant protein lactylation and metabolic dysregulation, representing a non‐histone lactylation event that links lactylation to translational control and metabolic dysregulation [94].

However, acyltransferases require lactyl‐CoA as the lactylation donor, while the intracellular concentration of lactyl‐CoA is far lower than that of acetyl‐CoA, raising doubts about whether these acyltransferases are the sole contributors to lactylation. Recent studies have identified an alternative, lactyl‐CoA‐independent mechanism, mediated by alanyl‐tRNA synthetase 1 and 2 (AARS1/2), which directly employs free lactate as a substrate to catalyze protein lactylation [91, 95]. This pathway appears to preferentially target non‐histone proteins and rapidly transmit metabolic signals to signaling and immune pathways. AARS1/2 first activate lactate by forming a lactyl‐AMP intermediate in an ATP‐dependent reaction, then transfer the lactyl group to lysine residues on target proteins, releasing AMP [91, 95]. Functionally, AARS1 translocates to the nucleus in response to elevated lactate, where it lactylates Yes‐associated protein (YAP, K90) and TEA domain family member (TEAD1, K108), activating the YAP‐TEAD transcriptional program and driving tumor growth [96]. In parallel, AARS2‐mediated lactylation of cyclic GMP‐AMP synthase (cGAS) inhibits innate immune signaling [95]. Notably, β‐alanine competes with lactate for AARS1 binding, suppressing p53 lactylation and potentiating the efficacy of chemotherapy [96]. Together, AARS1 and AARS2 function as bona fide lactyltransferases, particularly in non‐histone contexts, translating metabolic fluctuations into epigenetic and signaling responses that promote tumor adaptation.

The removal of protein lactylation is mediated by two major “eraser” enzyme families, including histone deacetylases (HDACs) and NAD^+^‐dependent sirtuins (SIRTs). Several members of these families, such as HDAC1–HDAC3 [97] and SIRT1–SIRT3 [98], have been shown to remove lactylation modifications from proteins. These delactylation activities apply to both histone and non‐histone substrates, suggesting a shared reversibility mechanism across chromatin and signaling proteins. Unlike “writers” and “erasers”, there are few studies on lactylation readers, and it is unknown how they mediate downstream effects. Current evidence indicates that the chromatin remodeler Brahma‐related gene 1 (BRG1) recognizes H3K18la and functions as a lactylation reader to promote cellular reprogramming and regulate gene expression related to epithelial‐mesenchymal transition [99]. In addition, double PHD fingers 2 (DPF2) is a lactylation reader of H3K14la, which can bind to H3K14la and facilitate oncogenic transcriptional activation [100]. Taken together, these recent studies define an emerging enzymatic landscape of lactylation, comprising “writers,” “erasers,” and “readers” (Table 1) [92, 94, 95, 97, 98, 99, 100, 101, 102, 103, 104, 105, 106, 107, 108, 109, 110, 111, 112, 113, 114, 115, 116, 185]. Understanding how these enzymes are temporally and spatially regulated across different tumor contexts will not only deepen insight into metabolic‐epigenetic crosstalk but also uncover new therapeutic opportunities to modulate disease‐associated gene expression.

**TABLE 1:** Key enzymes involved in protein lactylation.

Classification	Mechanism	Key enzymes	Major tumor types involved	Biological consequences	Therapeutic implications	References
Writers	Lactyl‐CoA‐independent (directly utilizes l ‐lactate to catalyze lysine lactylation)	AARS1	Not specified	Catalyzes non‐canonical lactylation of p53 → DNA binding and transcriptional activation	β‐alanine inhibits lactate binding to AARS1, global lacylation, and tumorigenesis	[107]
			Hepatocellular carcinoma	Lactylation of ASH2L → tumor angiogenesis	Not specified	[112]
			Gastric cancer	AARS1 lactylates and activates the YAP‐TEAD complex → cancer proliferation and angiogenesis	Not specified	[96]
			Bladder cancer	AARS1‐mediated lactylation of YTHDC1 modulates bladder cancer sensitivity to enfortumab vedotin	Not specified	[111]
			Not specified	Mediates BLM lactylation and promotes HR repair	Irinotecan reverses anthracycline resistance by inhibiting BLM lactylation	[140]
			Not specified	Associates with cGAS to suppress antitumor immunity	Not specified	[95]
		AARS2	Glioblastoma	Interacts with ACSS2 to promote histone lactylation via KAT2A, driving tumor growth and immune evasion	Combined ACSS2‐KAT2A‐blocking peptides and PD‐1 antibodies markedly inhibit tumor progression	[92]
			Not specified	Associates with cGAS to suppress antitumor immunity	Not specified	[95]
	Lactyl‐CoA‐dependent	p300/CBP	Not specified	Mediates lactylation of MRE11 → DNA repair and chemoresistance	Blocking MRE11 lactylation impairs HR and sensitizes cancer cells to cisplatin	[113]
		MYST family	Colorectal cancer	KAT8‐mediated lactylation of EEF1A2 →protein synthesis	Deletion or inhibition of KAT8 suppresses colorectal tumor growth	[94]
			Not specified	TIP60‐mediated lactylation of NBS1 →DNA repair and chemoresistance	Not specified	[106]
		GNAT family	Breast cancer	RCC2 lactylation at K124 promotes cell‐cycle progression and metastasis	Small‐molecule inhibitors targeting the RCC2 active site specifically block lactylation and reduce proliferation	[114]
			Lung adenocarcinoma	HAT promotes HR repair by catalyzing RPA1 lactylation	Not specified	[139]
Erasers	Zn^2^^+^‐dependent	HDAC1‐3	Hepatocellular carcinoma	Remove lysine lactylation from histone and non‐histone substrates	HDAC activation reduces pathological lactylation and may restore metabolic homeostasis	[97, 112]
	NAD^+^‐dependent	SIRT1‐3	Glioma stem cells	De‐lactylate histones and regulate metabolic gene expression	Potential targets for epigenetic therapy in tumors	[98, 115]
Readers	Not specified	BRG1	Not specified	Recognizes H3K18la to activate EMT‐related gene expression and cellular reprogramming	Not specified	[99]
	Not specified	DPF2	Not specified	Binds H3K14la to facilitate oncogenic transcriptional activation	Not specified	[100]

Abbreviations: AARS1, alanyl‐tRNA synthetase 1; AARS2, alanyl‐tRNA synthetase 2; ACSS2, acetyl‐CoA synthetase 2; ASH2L, absent, small, or homeotic discs 2‐like; BLM, Bloom syndrome protein; BRG1, Brahma‐related gene 1; CBP, CREB‐binding protein; cGAS, cyclic GMP‐AMP synthase; DPF2, double PHD fingers 2; EEF1A2, eukaryotic translation elongation factor 1A2; GNAT, GCN5‐related N‐acetyltransferase; HAT1, histone acetyltransferase 1; HDAC, histone deacetylase; HR, homologous recombination; KAT2A, histone acetyltransferase KAT2A; KAT8, histone acetyltransferase KAT8; MRE11, MRE11 homolog, double‐strand break repair nuclease; MYST, MOZ‐Ybf2/Sas3‐Sas2‐Tip60 family; NAD^+^, nicotinamide adenine dinucleotide; NBS1, Nijmegen breakage syndrome protein 1; PD‐1, programmed cell death protein 1; RCC2, regulator of chromosome condensation 2; RPA1, replication protein A1; SIRT, sirtuin; TEAD, TEA domain transcription factor; TIP60, histone acetyltransferase TIP60; YAP, Yes‐associated protein; YTHDC1, YTH domain–containing protein 1; Zn^2+^, Zinc ion.

### 3.2 Roles of Lactylation in Cancer Progression

**FIGURE 3:**
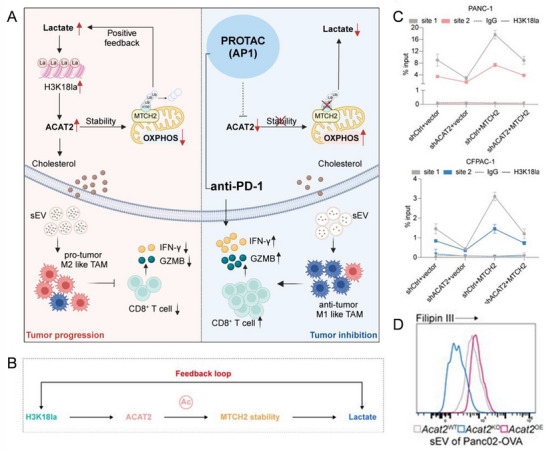
**Histone lactylation‐driven cholesterol metabolic loop in pancreatic cancer**. (A) Schematic illustration of the ACAT2/MTCH2/lactate positive feedback loop driven by histone lactylation, which promotes cholesterol‐mediated immunosuppressive reprogramming. (B) Diagram showing the positive feedback loop between H3K18la and ACAT2. (C) qChIP assay revealed H3K18la enrichment at the indicated promoters in ACAT2‐knockdown and MTCH2‐overexpressing PDAC cells. (D) Filipin III staining of BMDMs treated with sEVs derived from pancreatic cancer cells highlights cholesterol accumulation. Abbreviations: ACAT2, Acetyl‐CoA acetyltransferase 2; AP1, Proteolysis‐targeting chimera (PROTAC) compound AP1; CD8^+^ T, Cytotoxic T lymphocyte; CFPAC‐1, Capan‐1 pancreatic cancer cell line; GZMB, Granzyme B; H3K18la, Histone H3 lysine 18 lactylation; IFN‐γ, Interferon gamma; IgG, Immunoglobulin G; M1, Classically activated macrophage; M2, Alternatively activated macrophage; MTCH2, Mitochondrial carrier 2; OXPHOS, Oxidative phosphorylation; PD‐1, Programmed cell death protein 1; PANC‐1, Pancreatic cancer cell line 1; PROTAC, Proteolysis‐targeting chimera; sEV, Small extracellular vesicle; TAM, Tumor‐associated macrophage. Reproduced with permission [126]. 2025, BMJ Publishing Group Ltd.

Lactylation is detected across many cancers, and its regulatory roles in cancer biology are receiving growing attention [117, 118, 119]. Emerging evidence indicates that lactylation promotes cancer cell proliferation, stemness, invasion, metastasis, immune evasion, and drug resistance [120, 121]. Mechanistically, lactylation affects essential protein functions—particularly those involved in stemness maintenance, genome integrity, chromatin organization, transcriptional activation, metabolic activity, and immune modulation [120, 121]. In general, lactylation establishes a self‐reinforcing feedback loop that boosts cancer progression (Figure 3).

#### 3.2.1 Lactylation Modulates DNA Damage Repair and Genome Stability

**FIGURE 4:**
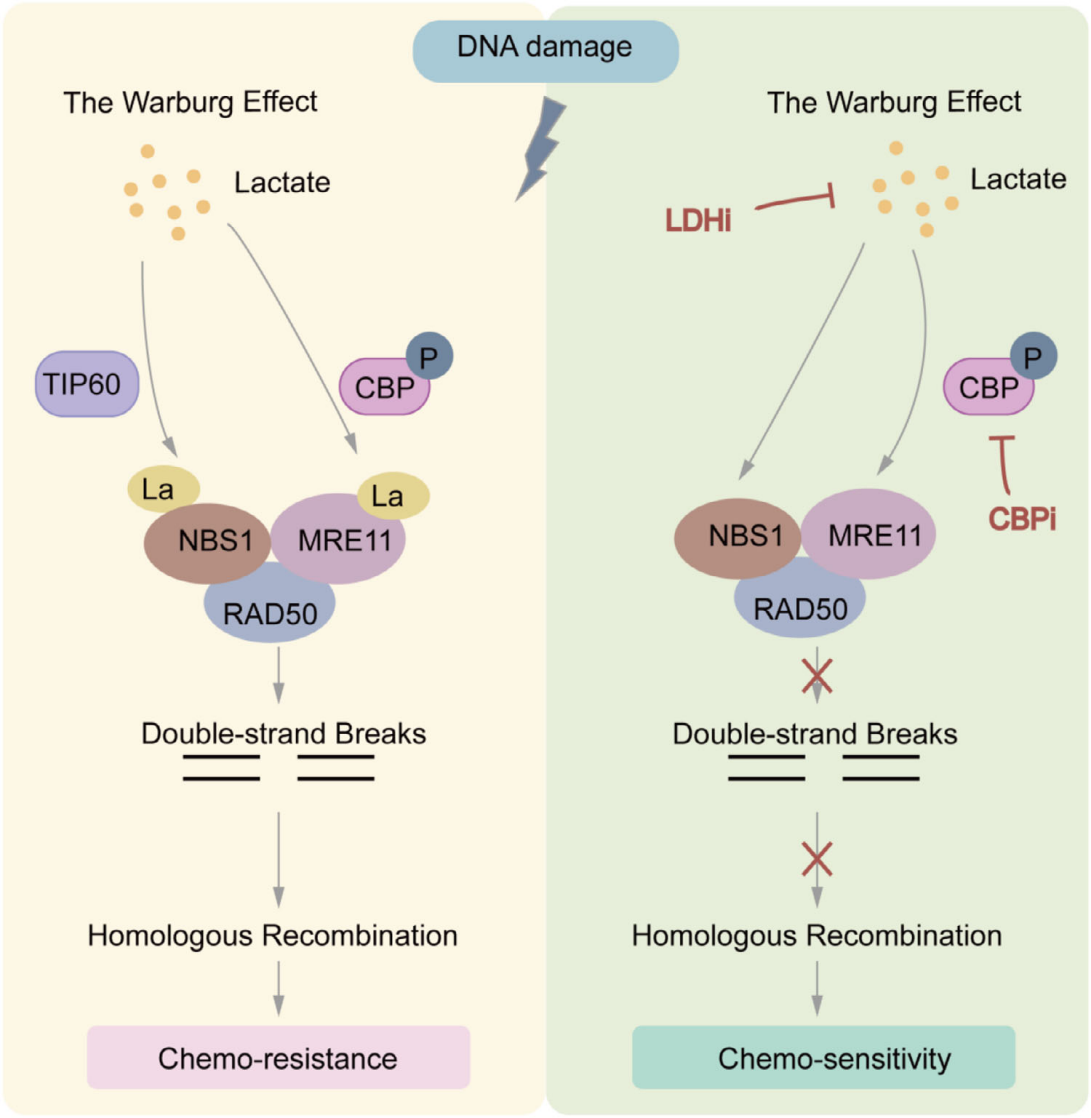
**Lactate‐driven DNA repair promotes chemotherapy resistance**. In chemo‐ or radio‐resistant tumor cells, lactate enhances TIP60‐mediated lactylation of NBS1, facilitating MRN complex formation and recruitment to DNA damage sites. Combination treatment with LDHi and CBPi disrupts this repair mechanism and reverses therapeutic resistance. Abbreviations: CBP, CREB‐binding protein; CBPi, CBP inhibitor; DNA, Deoxyribonucleic acid; La, Lactyl group; LDHi, Lactate dehydrogenase inhibitor; MRE11, Meiotic recombination 11 homolog; NBS1, Nijmegen breakage syndrome 1; P, Phosphorylation; RAD50, DNA repair protein RAD50; TIP60, Histone acetyltransferase TIP60.

Enhanced DNA damage repair is one of the key mechanisms by which cancer cells acquire chemoresistance, particularly through hyperactivation of homologous recombination (HR) pathways. The MRE11‐RAD50‐NBS1 (MRN) complex is indispensable for sensing DNA double‐strand breaks (DSBs) and initiating repair signaling. Recent studies identify lactylation as a critical regulator of this process (Figure 4) [138]. Specifically, NBS1 lactylation facilitates MRN complex assembly and recruitment to DNA damage sites, whereas MRE11 lactylation stabilizes the MRN complex and promotes DNA‐end resection—indicating a temporally coordinated role for lactylation in HR repair [106].

Moreover, lactylation modulates genome stability by targeting multiple HR factors. In lung adenocarcinoma, HAT1 acts as a lactyltransferase that induces RPA1 lactylation, enhancing its binding to single‐stranded DNA and the MRN complex, thereby accelerating HR repair; loss of HAT1 or its auto‐lactylation impairs HR and increases radiosensitivity [139]. Likewise, a global lactylome screen in anthracycline‐resistant tumors identified that BLM K24 lactylation prevents its ubiquitination, stabilizes BLM protein, and strengthens HR activity. Blocking the AARS1‐BLM lactylation axis reverses chemoresistance and restores sensitivity to DNA‐damaging therapy [140].

Clinically, high lactylation levels of NBS1 and MRE11 correlate with poor prognosis in patients receiving neoadjuvant chemotherapy, consistent with the notion that lactylation‐driven HR activation and genome stabilization contribute to treatment resistance [106]. These results collectively suggest that metabolic interventions targeting lactate production or utilization can enhance therapeutic efficacy by disrupting lactylation‐mediated DNA repair pathways [106].

#### 3.2.2 Lactylation Bridges Metabolic Rewiring and Epigenetic Regulation

Advances in mass spectrometry have revealed lactate as a critical metabolite that can influence gene regulation and signal transduction through chromatin remodeling and PTMs [163, 164]. Intracellular and extracellular lactate levels are key determinants of histone lactylation, whose abundance is tightly coupled to the balance between glycolysis and mitochondrial OXPHOS [21, 165, 166]. Although both lactate and acetyl‐CoA originate from the glycolytic end‐product pyruvate, their spatially separated production, cytosolic lactate versus mitochondrial acetyl‐CoA [164], means that the efficiency of pyruvate transport into mitochondria critically shapes cellular lactylation levels, subsequently influencing metabolic homeostasis. The correlation between histone Kla and intracellular lactyl‐CoA concentrations underscores the tight integration of cellular metabolism and epigenetic regulation in cancer biology [167].

Oncogenic signaling further strengthens glycolysis and lactate production, thereby enhancing lactylation. Activation of mTOR stimulates HIF1α and cellular myelocytomatosis oncogene (c‐Myc), two transcription factors that drive cancer cell metabolic reprogramming [169]. HIF1α promotes glycolytic gene expression and lactate production, whereas c‐Myc induces the expression of MCT1 and MCT4, facilitating lactate export and uptake, namely the lactate shuttle [169]. The transcription factor GLIS1 also contributes to metabolic reprogramming by binding to glycolytic gene promoters, increasing the production of both lactate and acetyl‐CoA, two critical metabolites that bridge metabolic rewiring and epigenetic regulation [170].

Additional oncogenic regulators reinforce this coupling. GLUT3, a high‐affinity glucose transporter, promotes lactylation by increasing LDHA expression [171]. STAT5, an oncogenic transcription factor, stimulates AKT signaling and GLUT1‐mediated glucose uptake [172]. In leukemia, STAT5 drives the transcription of hexokinase 1 (HK1), phosphofructokinase platelet isoform (PFKP), and pyruvate dehydrogenase A (PDHA), promoting aerobic glycolysis and inducing E3‐binding protein (E3BP) nuclear translocation, which in turn increases lactylation [150]. Likewise, pyruvate kinase M2 (PKM2) tetramerization in glioblastoma CSCs remodels glucose metabolism, resulting in lactate accumulation and elevated lactylation levels [162]. Collectively, these metabolic shifts converge to promote glycolysis and lactate production, acidifying the TME and establishing a pro‐lactylation metabolic state.

Lactylation, in turn, feeds back to modulate metabolic gene expression. Kla alters metabolic enzymes across nearly all dysregulated pathways associated with malignant transformation and progression [113, 143, 146, 156, 173, 174]. Many glycolytic enzymes are lactylation targets; for example, aldolase A (ALDOA) is lactylated at lysine 147 (K147), reducing its enzymatic activity and potentially forming a negative feedback mechanism that limits excessive lactate accumulation [175]. Histone lactylation also enhances the transcription of glycolytic and TCA cycle enzymes, as observed in non‐small cell lung cancer (NSCLC) cells [176]. Similarly, positive feedback between glycolysis and histone lactylation has been observed in Alzheimer's disease and pancreatic ductal adenocarcinoma [177, 178]. These findings indicate that lactylation not only results from metabolic reprogramming but also reinforces it through epigenetic activation of metabolic genes.

Hypoxia amplifies this glycolysis‐lactylation axis in cancer [179]. Serine hydroxymethyl transferase 2 (SHMT2), a key metabolic enzyme linked to cancer stemness, becomes upregulated and hyper‐lactylated under hypoxic conditions, enhancing its stability and glycolytic function [180]. Conversely, AXIN1 suppresses glycolysis and cancer stemness, but hypoxia‐induced lactylation of AXIN1 reverses this inhibition, promoting glycolytic and malignant phenotype of esophageal cancer cells [179]. Beyond nuclear and cytosolic proteins, mitochondrial proteins also undergo lactylation. In hypoxic mouse myocytes, AARS2 accumulation induces mitochondrial protein lactylation and suppresses OXPHOS [181]. Given the hypoxic and lactate‐rich conditions commonly observed in cancer, mitochondrial protein lactylation may contribute to cancer progression in a similar manner.

Besides, lactylation can interact with other PTMs, forming an intricate crosstalk among different epigenetic modifications [182, 183]. Lactate secreted by CAFs reprograms lipid metabolism, promoting lipid droplet formation and mobilization, and providing acetyl groups for histone acetylation [184]. Pharmacological inhibition of the bromodomain and extra‐terminal (BET) family, histone acetylation readers, reduces the expression of perilipin 2 (PLIN2), a key lipid droplet structural protein, disrupting lactate‐dependent lipid metabolic gene expression [184]. In hepatocellular carcinoma, acetylation of the pyruvate dehydrogenase complex component X (PDHX) has been identified as a novel mechanism driving cancer progression [186]. PDHX acetylation disrupts pyruvate dehydrogenase complex assembly, diverting glucose flux toward lactate production and aerobic glycolysis, thereby promoting H3K56 lactylation‐mediated gene activation and tumor growth [186].

In summary, aerobic glycolysis in cancer cells generates abundant lactate that fuels histone and/or other protein lactylation, tightly coupling metabolic rewiring with epigenetic regulation. Lactylation in turn reshapes cellular metabolism within the TME by modulating enzyme activity and gene expression. Notably, even transient epigenetic alterations are sufficient to establish persistent malignant states [187]. Targeting this metabolic‐epigenetic regulatory circuit represents a promising therapeutic approach. Further studies should delineate the mechanistic interplay between metabolic reprogramming and epigenetic perturbations to uncover potential vulnerabilities for cancer treatment.

#### 3.2.3 Lactylation Drives Therapeutic Resistance to Cancer Immunotherapy

Recent studies reveal that lactylation plays a pivotal role in cancer immunotherapy resistance by reprogramming lipid metabolism, modifying immune cell function, and shaping an immunosuppressive TME. In hepatocellular carcinoma, histone lactylation enhances lipid metabolic reprogramming [122]. Besides, lactylation of apolipoprotein C‐II (APOC2) promotes extracellular lipid degradation, drives Treg accumulation, and induces immunotherapy resistance [123]. Furthermore, elevated glycolytic flux and lactate accumulation intensify lactylation in CSCs from glioma and liver cancer [115, 124], supporting a metabolic foundation for immune evasion.

Lactylation is widespread in immune cells, reshaping the TME toward an immunosuppressive state. In macrophages, lactate‐driven histone lactylation promotes M2 polarization [125]. In pancreatic cancer, histone lactylation at the ACAT2 promoter enhances cholesterol biosynthesis and its export via sEVs, reinforcing M2 macrophage differentiation (Figure 3) [126]. Clinical imaging using 18F‐fluorodeoxyglucose (18F‐FDG) has shown that tumors with globally elevated protein lactylation exhibit poor immunotherapy responses. Mechanistic studies demonstrate that non‐histone lactylation of endosulfine alpha (ENSA) at lysine 63 (K63la) activates STAT3‐CCL2 signaling, resulting in TAM recruitment and upregulating immunosuppressive genes, including *CCL2, ARG1, S100A9*, and *IL10*, ultimately suppressing CTL infiltration and impairing PD‐1 blockade efficacy [127].

Lactate released by cancer cells also induces histone lactylation‐dependent expression of nuclear protein 1 (NUPR1) in macrophages, promoting M2 polarization and upregulation of immune checkpoints, including PD‐L1 and signal regulatory protein alpha (SIRPα) [128]. These changes lead to CD8^+^ T‐cell exhaustion and weaken immunotherapy responsiveness [128]. In activated CD8^+^ T cells, histone marks H3K9la and H3K18la are enriched and act as transcriptional initiators for key effector genes [129]. However, in head and neck squamous cell carcinoma, excessive H3K9la is linked to poor immunotherapy response, as H3K9la stimulates IL‐11 secretion from cancer cells, causing CD8^+^ T‐cell exhaustion [130]. Lactylation also reshapes other immune cells. Hypoxia‐induced histone lactylation in CD71^+^ neutrophils upregulates ARG1, a critical immunosuppressive enzyme that inhibits T‐cell function [131]. In natural killer (NK) cells, elevated Kla is associated with impaired NAD^+^ metabolism, mitochondrial fragmentation, and diminished cytotoxic activity [132]. In Tregs, lactylation upregulates tumor necrosis factor receptor 2 (TNFR2) expression, which correlates with poor prognosis in patients with malignant pleural effusion [133].

Within cancer cells, lactylation further strengthens immune evasion by directly regulating immune checkpoint pathways. Lactylation activates transcription of B7‐H3, an immune checkpoint molecule whose overexpression suppresses CD8^+^ T‐cell infiltration and cytotoxicity, facilitating immune escape and cancer progression [134]. Lactylation also induces PD‐L1 expression; in NSCLC, H3K18la has been shown to enhance immune evasion by upregulating PD‐L1 [135]. Furthermore, lysyl oxidase secreted by CAFs promotes PD‐L1 expression through histone lactylation [136]. Beyond transcriptional control, PD‐L1 itself undergoes non‐histone lactylation that stabilizes the protein and delays its degradation, thereby undermining the efficacy of PD‐1/PD‐L1 immune checkpoint blockade therapies [137]. Interestingly, dietary interventions, such as a serine/glycine‐restricted diet, have shown potential in counteracting this effect and potentiate immunotherapeutic responses [137].

In summary, lactylation orchestrates cancer initiation and progression through diverse molecular pathways, reflecting the broad functional impact of lactate‐derived modifications (Table 2) [92, 93, 94, 106, 113, 115, 123, 124, 135, 141, 142, 143, 144, 145, 146, 147, 148, 149, 150, 151, 152, 153, 154, 155, 156, 157, 158, 159, 160, 161, 162]. The discovery of lactylation as a PTM has expanded the known biological functions of lactate metabolism and established a mechanistic link between metabolic reprogramming, epigenetic regulation, immune evasion, and treatment resistance [189].

**TABLE 2:** The regulatory effect of lactylation on cancers.

Cancer types	Lactylation sites	Lactyltransferase (Writers)	Delactylase (Erasers)	Function and mechanisms	References
Endometrial carcinoma	H3K18la	Not specified	Not specified	Promotes cancer progression	[141]
Bladder cancer	H3K18la	Not specified	Not specified	Promotes cancer progression via regulation of LCN2 transcription	[142]
Anaplastic thyroid cancer (ATC)	H4K12la	Not specified	Not specified	BRAFV600E oncogene promotes proliferation via metabolite‐mediated histone lactylation	[143]
Neuroblastoma	H4K5la	Not specified	Not specified	Promotes cancer progression	[120]
Glioma	H3K18la	Not specified	Not specified	Regulates BNIP3‐dependent mitophagy‐mediated metabolic reprogramming, enhancing proliferation and invasion	[145]
Colorectal cancer (CRC)	EEF1A2K408	KAT8	Not specified	Enhances translation elongation and protein synthesis, promoting cancer development	[94]
Colorectal cancer (CRC)	β‐catenin	Not specified	Not specified	Promotes proliferation and stemness via WNT signaling pathway	[146]
Hepatocellular carcinoma (HCC)	YAP‐K90la	Not specified	Not specified	Promotes cancer cell proliferation and stemness	[147]
Glioma	PTBP1‐K436	Not specified	SIRT1	Promotes cancer cell stemness	[115]
Hepatocellular carcinoma (HCC)	H3K56la and ALDOA‐K230/322	Not specified	Not specified	Promotes cancer cell stemness	[124]
Colorectal cancer (CRC)	H3K18la	Not specified	Not specified	Accelerates liver metastasis	[148]
Hepatocellular carcinoma (HCC)	ABCF1‐K430	Not specified	Not specified	Promotes tumor growth and lung metastasis	[149]
Acute myeloid leukemia (AML)	H4K5	Not specified	Not specified	Drives immune suppression	[150]
Glioblastoma	H3K18la	Not specified	Not specified	Upregulates CD39, CD73, and CCR8, forming an immunosuppressive tumor microenvironment	[151]
Multiple cancers	H3K18la	Not specified	Not specified	Promotes cancer immune escape	[152]
Colorectal cancer (CRC)	METTL‐ K281, K345	Not specified	Not specified	Promotes m6A‐mediated immunosuppression in tumor‐infiltrating myeloid cells	[153]
Liver cancer	MOESIN‐ Lys72	Not specified	Not specified	Modulates TGF‐β signaling in Treg cells to promote progression	[154]
Bladder cancer	H3K18la	Not specified	Not specified	Enhances PRKN expression, promoting mitophagy and M2 macrophage polarization	[155]
Multiple cancers	H3K18la	ACSS2‐KAT2A	Not specified	Promotes cancer immune evasion	[92]
Non–small cell lung cancer (NSCLC)	H3K18la	Not specified	Not specified	Promotes cancer immune evasion	[135]
Bladder cancer	H3K18la	Not specified	Not specified	Drives cisplatin resistance via YBX1 and YY1 transcription factors	[156]
Colorectal cancer (CRC)	H3K18la	Not specified	Not specified	Increases expression of RUBCNL, enhancing autophagy, survival, and therapeutic resistance	[157]
Glioblastoma	H3K9la	Not specified	Not specified	Confers temozolomide resistance	[158]
Colorectal cancer (CRC)	H4k12la	Not specified	Not specified	Increases ABC transporter expression, reducing proliferation and conferring chemotherapy insensitivity	[159]
Multiple cancer cell lines	MRE11‐ K673	CBP	SIRT1/2	Promotes homologous recombination repair and chemotherapy resistance	[113]
Hepatocellular carcinoma (HCC)	NSUN2 K508R	NAA10	Not specified	Drives resistance to ferroptosis	[160]
Glioma	H3K18la	GTPSCS/p300	Not specified	Promotes growth and radioresistance	[93]
Hepatocellular carcinoma (HCC)	IGF2BP3	Not specified	Not specified	Mediates serine metabolism reprogramming and promotes lenvatinib resistance	[161]
Glioblastoma	XRCC1‐K247	Not specified	Not specified	Promotes drug resistance	[162]
Non–small cell lung cancer (NSCLC)	APOC2‐K70	Not specified	Not specified	Causes immune therapy resistance and metastasis	[123]
Multiple cancers	NBS1‐K388	Not specified	Not specified	Promotes DNA repair and chemotherapy resistance	[106]
Gastric cancer	METTL16‐K229	AARS1 / AARS2	SIRT2	Induces cuproptosis via m6A modification of *FDX1* mRNA	[282]
Lung cancer	AIM2	Not specified	Not specified	Reduces ferroptosis via regulation of ACSL4 and STAT5B	[283]
Breast cancer	H3K18la	Not specified	Not specified	High glycolysis state upregulates c‐Myc–SRSF10 axis via H3K18la	[166]
Pancreatic ductal adenocarcinoma (PDAC)	H3K18la	EP300	HDAC2	Glycolysis–H3K18la–TTK/BUB1B positive feedback loop promotes cancer progression	[178]
Lung adenocarcinoma	RPA1‐K88/163/167/267la	HAT1	Not specified	Enhances RPA1 binding to ssDNA and recruitment of MRN/HR factors, promoting HR repair and radioresistance	[139]
Multiple cancers	BLM‐K24la	AARS1	Not specified	Stabilizes BLM by preventing ubiquitination, enhances BLM interaction with DNA repair factors, improves HR repair, and drives anthracycline chemoresistance	[140]
Multiple cancers	p53‐K120, K139	AARS1	Not specified	Reduces tumor suppressor activity of p53 in vitro and in vivo	[107]

Abbreviations: AARS1, Alanyl‐tRNA synthetase 1; AARS2, Alanyl‐tRNA synthetase 2; ABCF1, ATP‐binding cassette sub‐family F member 1; ABC, ATP‐binding cassette; ACSL4, Acyl‐CoA synthetase long‐chain family member 4; AIM2, Absent in melanoma 2; ALDOA, Aldolase A; AML, Acute myeloid leukemia; AP, Autophagy protein; APOC2, Apolipoprotein C‐II; ATC, Anaplastic thyroid cancer; BLM, Bloom syndrome protein; BNIP3, BCL2/adenovirus E1B 19 kDa protein‐interacting protein 3; BRAFV600E, B‐Raf proto‐oncogene, serine/threonine kinase V600E; CBP, CREB‐binding protein; CCR8, C‐C chemokine receptor type 8; CRC, Colorectal cancer; DNA, Deoxyribonucleic acid; EEF1A2, Eukaryotic translation elongation factor 1A2; EP300, E1A binding protein p300; FDX1, Ferredoxin 1; HAT1, histone acetyltransferase 1; HDAC2, Histone deacetylase 2; HCC, Hepatocellular carcinoma; HR, homologous recombination; IGF2BP3, Insulin‐like growth factor 2 mRNA‐binding protein 3; KAT2A, Histone acetyltransferase KAT2A; KAT8, Histone acetyltransferase KAT8; LCN2, Lipocalin 2; METTL3, Methyltransferase‐like protein 3; METTL16, Methyltransferase‐like protein 16; MRE11, MRE11 homolog double‐strand break repair nuclease; m6A, N6‐methyladenosine; NAA10, N(alpha)‐acetyltransferase 10; NBS1, Nijmegen breakage syndrome 1; NSCLC, Non–small cell lung cancer; NSUN2, NOP2/Sun RNA methyltransferase 2; p300, E1A binding protein p300; PDAC, Pancreatic ductal adenocarcinoma; PRKN, Parkin RBR E3 ubiquitin protein ligase; PTBP1, Polypyrimidine tract‐binding protein 1; RPA1, replication protein A1; RUBCNL, Rubicon‐like protein; SIRT1, Sirtuin 1; SIRT2, Sirtuin 2; SRSF10, Serine/arginine‐rich splicing factor 10; STAT5B, Signal transducer and activator of transcription 5B; TGF‐β, Transforming growth factor beta; TTK, Threonine tyrosine kinase; Treg, Regulatory T cell; WNT, Wingless/Integrated signaling pathway; XRCC1, X‐ray repair cross‐complementing protein 1; YAP, Yes‐associated protein; YBX1, Y‐box‐binding protein 1; YY1, Yin Yang 1.

## 4 Targeting Lactate and Lactylation in Cancer Therapy

**FIGURE 5:**
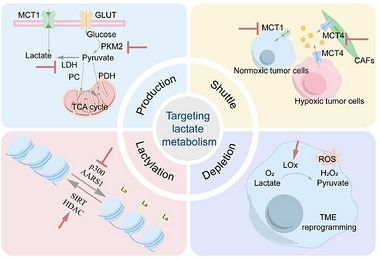
**Clinical application strategies targeting lactate metabolism**. The lactate metabolism pathway involves various steps of lactate production, shuttling, and depletion. Clinical application strategies are formulated by targeting key enzymes involved in lactate metabolic processes. Besides, lactylation can also be regulated by targeting lactyltransferase and delactylase. Abbreviations: AARS1, Alanyl‐tRNA synthetase 1; CAF, Cancer‐associated fibroblast; GLUT, Glucose transporter; HDAC, Histone deacetylase; H_2_O_2_, Hydrogen peroxide; La, Lactyl group; LDH, Lactate dehydrogenase; LOx, Lactate oxidase; MCT1, Monocarboxylate transporter 1; MCT4, Monocarboxylate transporter 4; O_2_, Oxygen; PC, Pyruvate carboxylase; PDH, Pyruvate dehydrogenase; PKM2, Pyruvate kinase M2; p300, E1A‐binding protein p300; ROS, Reactive oxygen species; SIRT1, Sirtuin 1; TCA, Tricarboxylic acid cycle.

Given the pivotal role of lactate and lactylation in shaping the TME, therapeutic strategies targeting this axis have emerged as a major focus of translational research. Elevated lactate levels not only reflect tumor metabolic reprogramming but also serve as potential biomarkers of malignancy. Accordingly, interventions aimed at modulating lactate production, transportation, utilization, and lactylation hold substantial therapeutic promise (Figure 5).

### 4.1 Clinical Potential of Lactate and Lactylation

Within primary tumors, lactate accumulation has considerable clinical relevance, as its concentration correlates strongly with disease stage, metastatic potential, recurrence risk, and overall survival, making it a candidate biomarker for cancer diagnosis and prognosis. Molecular signatures related to lactate metabolism, lactylation, and lactate‐associated long noncoding RNAs (lncRNAs) have shown prognostic value in risk stratification, therapeutic selection, and prediction of responses to immunotherapy [190, 191, 192, 193, 194, 195].

Accurate lactate quantification, both in vitro and through minimally invasive in vivo or in situ approaches, is essential for clinical application. Leveraging the specificity of lactate oxidation, lactate oxidase (LOx)‐based biosensors have been developed to detect cancer‐associated lactate. These biosensors monitor H_2_O_2_ generation, O_2_ consumption, pH variation, and/or electrochemical readouts, depending on the detection modality [196]. Such sensors have enabled real‐time recording of lactate‐dependent voltammetric signals in tumor‐bearing mice [197]. In parallel, metabolic imaging of lactate dynamics in tumors offers a promising avenue for noninvasive diagnosis. Overall, biochemical and imaging‐based detection of lactate holds great potential to inform cancer staging, achieve therapeutic monitoring, and predict outcomes in diverse cancers.

### 4.2 Targeting Lactate in Cancer Therapy

Given its crucial role in cancer progression, lactate metabolism offers multiple targets for therapeutic intervention. Strategies designed to inhibit lactate production, block its transport, and/or modulate its utilization within the TME have shown promising preclinical and clinical efficacy (Table 3) [198, 199, 200, 201, 202, 203, 204, 205, 206, 207, 208, 209, 210, 211, 212, 213, 214, 215, 216, 217, 218, 219, 220, 221, 222, 223, 224, 225, 226, 227, 228, 229].

**TABLE 3:** Research on targeting lactic acid metabolism for cancer treatment.

Cancer types	Lactate modulator/ Chemical/ Inhibitors	Targets	Function and efficacy	References
a. Inhibition of lactate production				
Multiple cancer cell lines	MDP, DDP	LDH	Inhibits lactate production and transport and disrupts tumor vasculature	[198]
Breast cancer	PCGF MPN	LDH	Remodels TME in murine breast cancer; elicits anti‐tumor immune responses	[199]
Pancreatic cancer cell lines	LDH PROTAC degrader	LDH	Suppresses cancer cell proliferation	[200]
Multiple cancer cell lines	Indoles	LDHA	Induces apoptosis and inhibits cancer cell growth	[201]
Colorectal cancer and liver metastases	SHK@HA‐MPDA	PKM2	Reduces MDSC migration; reverses EMT	[202]
Anaplastic thyroid carcinoma	Shikonin	PKM2	Promotes ferroptosis; inhibits glycolysis	[203]
Pancreatic ductal adenocarcinoma	FV‐429	PKM2	Inhibits glycolysis; induces mitochondrial apoptosis in PDAC	[204]
b. Targeting lactate transport				
Anaplastic thyroid cancer	Acriflavine, syrosingopine, AZD3965	MCT4	Inhibits lactate shuttle; reduces cancer cell proliferation and glycolysis in ATC cells	[205]
Multiple cancer cell lines	LC	MCT1	Relocates MCT1 to the inner mitochondrial membrane, diverting lactate to mitochondria for CD8^+^ T cell energy	[206]
Hepatocellular carcinoma	VB124	MCT4	Enhances T cell infiltration and function; augments immunotherapy in HCC	[207]
Non‐small cell lung cancer	FC	MCT4/CD147	Blocks MCT4/CD147‐mediated lactate efflux; suppresses NSCLC progression	[208]
Urothelial bladder cancer	AZD3965	MCT1	Exhibits anti‐cancer activity in tumors with low levels of MCT4	[209]
B‐cell cancer	AZD3965, AR‐C155858	MCT1	Selectively inhibits B‐cell cancer growth; CAR‐T cells remain unaffected	[210]
Breast cancer	BGal2C	MCT4	Disrupts lactate transport	[211]
Metastatic cancer	PM‐Lipo	MCT4	Prevents cancer metastasis	[212]
Breast cancer	miR‐425‐5p	MCT4	Impairs CAF‐mediated angiogenesis, migration, and cancer cell viability/proliferation	[213]
c. Inducing lactate depletion				
Multiple cancers	n(LOx)	LOx	Reduces lactate; releases H_2_O_2_; alleviates immunosuppression; enhances immunotherapy efficacy	[214]
Multiple cancers	Co_4_N/C NEs	LOx‐mimic	Reverses high‐lactate, immunosuppressive TME	[215]
Breast cancer cell line	LOx+ SIRPα genome‐editing plasmids +MOFs	LOx	Repolarizes TAMs; improves immunotherapy	[216]
Breast cancer cell line	PNDPL	LOx	Depletes lactate; photothermal therapy; blocks IDO‐mediated immune escape	[217]
Colon carcinoma cell line	AaLS/LOx/CAT	LOx	Inhibits cancer growth	[218]
Melanoma cell line	LOx/vSIRPα conjugates	LOx	Induces necroptosis via complete lactate depletion	[219]
Breast cancer	PFOB@F127@PDA	LOx	Reverses immunosuppression	[220]
Breast cancer cell line	Metformin + LOx@MF	LOx	Inhibits angiogenesis and cancer cell proliferation	[221]
Spinal‐metastasized tumors	MP@AL	LOx	Remodels immunosuppressive TME; boosts immunotherapy	[222]
Breast cancer cell line	ILH	LOx	Reverses immunosuppression	[223]
Clear cell renal cell carcinoma	mCGYL‐LOx	LOx	Blocks immune escape	[224]
Pancreatic cancer	SPN_LCu_	LOx	Promotes cuproptosis	[225]
Neuroblastoma	LOx/HRP‐aZIF	LOx	Promotes ferroptosis	[226]
Multiple cancer cell lines	LOx@ZIF‐8@MPN	LOx	Enhances intratumoral T‐cell responses; promotes anti‐PD‐1 immunotherapy	[227]
Multiple cancers	PLNR840	LOx	Synergizes with photo‐immunotherapy	[228]
Multiple cancer cell lines	LOx/CAT + siVEGF	LOx	Inhibits angiogenesis and cancer cell proliferation	[229]

Abbreviations: AaLS, Aquifex aeolicus lumazine synthase; ATO, Atovaquone; ATC, Anaplastic thyroid cancer; AZD3965, Monocarboxylate transporter 1 inhibitor AZD3965; BGal2C, Beta‐galactosidase 2C; CAF, Cancer‐associated fibroblast; CAR T, Chimeric antigen receptor T cell; CAT, Catalase; Co_4_N/C NEs, Cobalt nitride/carbon nanoenzymes; DDP, c,c,t‐[Pt(NH_3_)_2_Cl_2_(OCOCH_2_C_6_H_4_NHC_6_H_3_Cl_2_)_2_]; DIDS, 4,4′‐Diisothiocyanatostilbene‐2,2′‐disulphonic acid; EMT, Epithelial–mesenchymal transition; FC, Formosanin C; FV‐429, Flavonoid derivative FV‐429; GF, Glucose–fructose; HCC, Hepatocellular carcinoma; HRP, Horseradish peroxidase; IDO, Indoleamine 2,3‐dioxygenase; ILH, A dual enzyme‐driven cascade reactions platform; LDH, Lactate dehydrogenase; LDHA, Lactate dehydrogenase A; LDHB, Lactate dehydrogenase B; LC, Lithium carbonate; LOx, Lactate oxidase; mCGYL, Cell membrane modifying CuO@Gd2O3yolk‐like particles; MCT1, Monocarboxylate transporter 1; MCT4, Monocarboxylate transporter 4; MDP, c,c,t‐[Pt(NH_3_)_2_Cl_2_(OCOCH_2_C_6_H_4_NHC_6_H_3_Cl_2_)(OH)]; MDSC, Myeloid‐derived suppressor cell; MF, MIL‐101 (Fe) nanoparticles; miR‐425‐5p, MicroRNA‐425‐5p; MnO_2_, Manganese dioxide; MOF, Metal–organic framework; MP@AL, Cancer cell membrane‐encapsulated pH‐responsive nitric oxide‐releasing biomimetic nanosystem; MPN, Metal–phenolic network; MPC, Mitochondrial pyruvate carrier; n(LOx), Lactate oxidase nanocapsules; NSCLC, Non–small cell lung cancer; PCGF, PEG‐Ce6‐CA‐GF‐Fe^2+^ MPNs; PD‐1, Programmed cell death protein 1; PDAC, Pancreatic ductal adenocarcinoma; PFOB, Perfluorooctyl bromide; PKM2, Pyruvate kinase M2; PM‐Lipo, pH‐sensitive liposome; PNDPL, PtBi/NLG919@DSPE‐PEG‐LOx; PROTAC, Proteolysis targeting chimera; SHK@HA‐MPDA, Shikonin‐loaded hyaluronic acid‐modified mesoporous polydopamine nanoparticles; siVEGF, Small interfering RNA targeting VEGF; SIRPα, Signal regulatory protein alpha; SPN_LCu_, Semiconducting polymer nanoreactor with copper; TAM, Tumor‐associated macrophage; TME, Tumor microenvironment; VB124, MCT4 inhibitor VB124; vSIRPα, Variant signal regulatory protein alpha; ZIF, Zeolitic imidazolate framework.

#### 4.2.1 Inhibition of Lactate Production

Reducing lactate accumulation in the TME by blocking its synthesis typically involves targeting key glycolytic enzymes. Elevated LDH expression strongly correlates with poor prognosis in many cancers [230], and LDH silencing has shown potent tumor‐suppressive effects [231]. Multiple LDH inhibitors, including oxalate, gossypol, FX11, galloflavin, and GNE140, demonstrate anti‐cancer efficacy in preclinical studies [232, 233, 234]. Mechanistically, LDH inhibitors suppress lactate production through the following routes: (1) downregulation of LDH expression; (2) competitive inhibition of LDHA active site by pyruvate; (3) blockade of the LDHA NADH‐binding site; and (4) promotion of LDHA degradation via the ubiquitin‐proteasome system [29, 198, 200, 233, 234].

Notably, certain clinically approved drugs, such as tucatinib and ceritinib, used in breast and lung cancer, can noncompetitively inhibit LDHB, highlighting opportunities to repurpose or optimize LDHB‐selective inhibitors [235]. However, LDHA inhibition may not be universally beneficial for all cancer types. In cervical cancer, LDHA knockout under energy‐stress conditions activates AMPK signaling, paradoxically promoting cancer cell survival and proliferation in vitro [236]. Furthermore, pyruvate kinase (PK), the terminal rate‐limiting enzyme in glycolysis, also represents a therapeutic target. Inhibiting tetrameric pyruvate kinase M2 (PKM2) reduces ATP and lactate production, thereby augmenting anti‐tumor immune activation [202]. Collectively, genetic and pharmacological inhibition of glycolytic enzymes involved in lactate synthesis shows promise in restraining tumor growth.

#### 4.2.2 Disruption of Lactate Transport

Lactate transport is primarily mediated by MCTs, especially MCT1 and MCT4. Strategies to disrupt MCT function include gene knockout, siRNA‐mediated silencing [237], direct pharmacological inhibition [205], and interference with the MCT chaperone CD147 [208]. MCT1 inhibition triggers apoptosis in hypoxic tumor cells that depend heavily on glucose due to metabolic competition with normoxic cells [169]. Conversely, MCT4 inhibition impairs lactate efflux, leading to intracellular lactate accumulation, cell cycle arrest, apoptosis, and increased reactive oxygen species (ROS) production [238].

Several natural and synthetic small‐molecule MCT inhibitors, such as quercetin, phloretin, and α‐cyano4‐hydroxycinnamate (CHC), have shown efficacy in preclinical cancer models [11, 169]. AZD3965, a specific MCT1 inhibitor, has demonstrated anti‐cancer activity in malignancies characterized by high MCT1 but low MCT4 expression [209], and has undergone phase I clinical trials in lymphoma and advanced solid tumors [239, 240]. The perivascular localization of MCT1‐expressing cancer cells makes MCT1 an accessible pharmaceutical target [241], although high MCT4 co‐expression can dampen therapeutic efficacy.

Because MCTs also play essential roles in normal physiology, systemic inhibition carries a significant risk of toxicity. Studies using MCT‐deficient mouse models have revealed that MCT1 loss impairs axonal myelination and neuronal survival, MCT2 deficiency compromises memory formation, and MCT3 ablation leads to visual dysfunction [242]. In early‐phase clinical trials of AZD3965, the most common adverse events included electroretinographic changes (retinopathy), nausea, fatigue, anorexia, and constipation, with occasional reports of elevated cardiac troponin levels and urinary ketones [11, 240]. In a phase I clinical trial of AZD3965 (NCT01791595; completed), a patient developed refractory hyperlactatemic acidosis following the first dose [243]. This dose‐limiting toxic event directly led to the suspension of trial recruitment, highlighting the risks associated with systemic inhibition of MCT1‐mediated lactate metabolism in normal tissues. These findings demonstrate that blocking lactate transport can disrupt essential physiological processes, underscoring the need for tumor‐selective delivery strategies and cautious clinical evaluation of MCT‐targeted therapies.

Besides, blocking MPC activity can disrupt lactate utilization. The MPC inhibitor 7ACC2 prevents mitochondrial pyruvate import, indirectly blocking extracellular lactate metabolism [241]. Compared with MCT inhibition, MPC blockade offers added benefits by preventing compensatory glucose consumption, inducing cytotoxicity, and reducing oxygen utilization, sensitizing tumors to radiotherapy [241]. Both in vitro and in vivo, inhibition of lactate transport reduces malignancy and enhances susceptibility to metabolic or immune‐based therapies.

#### 4.2.3 Induction of Lactate Depletion

Under normoxic conditions, LOx catalyzes the conversion of lactate to produce pyruvate and H_2_O_2_, simultaneously depleting lactate in the TME and generating cytotoxic ROS [197]. This dual action reverses lactate‐induced immunosuppression [214, 216], inhibits angiogenesis [244], and directly damages cancer cells through H_2_O_2_‐derived oxidative stress [216]. Moreover, H_2_O_2_ can be further converted into highly reactive hydroxyl radicals (·OH) via Fenton or Fenton‐like reactions, redox processes in which transition metal ions catalyze the decomposition of H_2_O_2_ to generate ·OH, amplifying cancer cell killing [245].

Because lactate is ubiquitous, systemic administration of high‐dose LOx risks severe off‐target toxicity to normal tissues and organs [218]. To mitigate this, current treatment strategies emphasize localized and sustained LOx delivery [246]. Delivery platforms include nanocomposites [214, 217, 218, 245, 246, 247, 248], metal‐organic frameworks [216, 249], and pH/lactate‐responsive multilayer films that degrade under tumor‐mimetic extracellular conditions (20 mM lactate, pH 6.5) [250]. Incorporating tumor‐targeting ligands within LOx carriers prolongs intratumoral retention and improves therapeutic efficacy [219]. Artificial nanoenzymes that mimic LOx catalytic activity have also emerged as promising alternatives [215].

However, hypoxia in the TME limits LOx activity by depleting oxygen, its essential substrate. To overcome this limitation, oxygen‐generating agents such as MnO_2_ [248] or catalase [218] that promote H_2_O_2_ decomposition are co‐delivered to maintain enzymatic function. Overall, lactate depletion strategies centered on LOx‐mediated oxidation have advanced through improved delivery systems that reduce systemic toxicity and enable combinatorial designs. Combining LOx with complementary modalities, including Fenton reagents [245], oxygen‐generating systems, and/or gene‐editing tools [216, 229], has shown synergistic anti‐cancer potential. Despite these advances, current lactate‐inhibiting and LOx‐based therapies face limitations, such as transient efficacy and incomplete tumor selectivity, which impede clinical translation and require further refinements.

### 4.3 Targeting Lactylation in Cancer Therapy

As discussed above, lactate and acetyl‐CoA are mainly generated from pyruvate metabolism in different cellular compartments and serve as substrates for Kla and acetylation, respectively. Their relative abundance determines the balance between these two epigenetic modifications. Since both processes share the same “writer” enzyme p300 [164], differences in enzymatic kinetics and substrate competition suggest that fluctuations in the lactylation‐acetylation balance can profoundly influence cell fate during malignant transformation.

Inhibiting glycolysis reduces Kla, whereas drugs targeting mitochondrial function tend to increase it [164]. Beyond modulating lactate metabolism and transport via enzymes such as LDH, MCT, and PKM2 [251], direct targeting of the enzymatic machinery involved in lactylation itself presents additional therapeutic opportunities. For instance, alanine competes with lactate for binding to AARS1, a lactate sensor, thereby preventing p53 lactylation and enhancing chemosensitivity [107]. Similarly, SIRT1 functions as an HDAC at H3K18, disrupting the H19/glycolysis/H3K18la positive feedback loop [252]. HDACs also act as “erasers” of lactylation, and several clinically approved HDAC inhibitors, including panobinostat, romidepsin, and vorinostat, may possess unrecognized potential for therapeutic modulation of lactylation [164].

Despite strong preclinical rationale, translating lactate‐ and lactylation‐targeted strategies into clinical oncology faces substantial challenges. Metabolic ubiquity and tissue heterogeneity complicate therapeutic selectivity, while limited understanding of enzyme kinetics and substrate specificity hinders rational drug design. Key lactylation enzymes, including AARS1/2 and KAT family members, exhibit overlapping acyltransferase activities and context‐dependent substrate preferences. Without detailed structural and kinetic characterization, developing highly selective small‐molecule inhibitors is difficult, increasing the risk of off‐target effects [91]. Moreover, reliable biomarkers of lactate accumulation, enzyme activity, or Kla levels in tumor biopsies and body fluids are still lacking. Establishing such indicators will be essential for evaluating drug responses and guiding the clinical translation of lactylation‐targeted therapies.

### 4.4 Targeting Lactate and Lactylation in Cancer Immunotherapy

**FIGURE 6:**
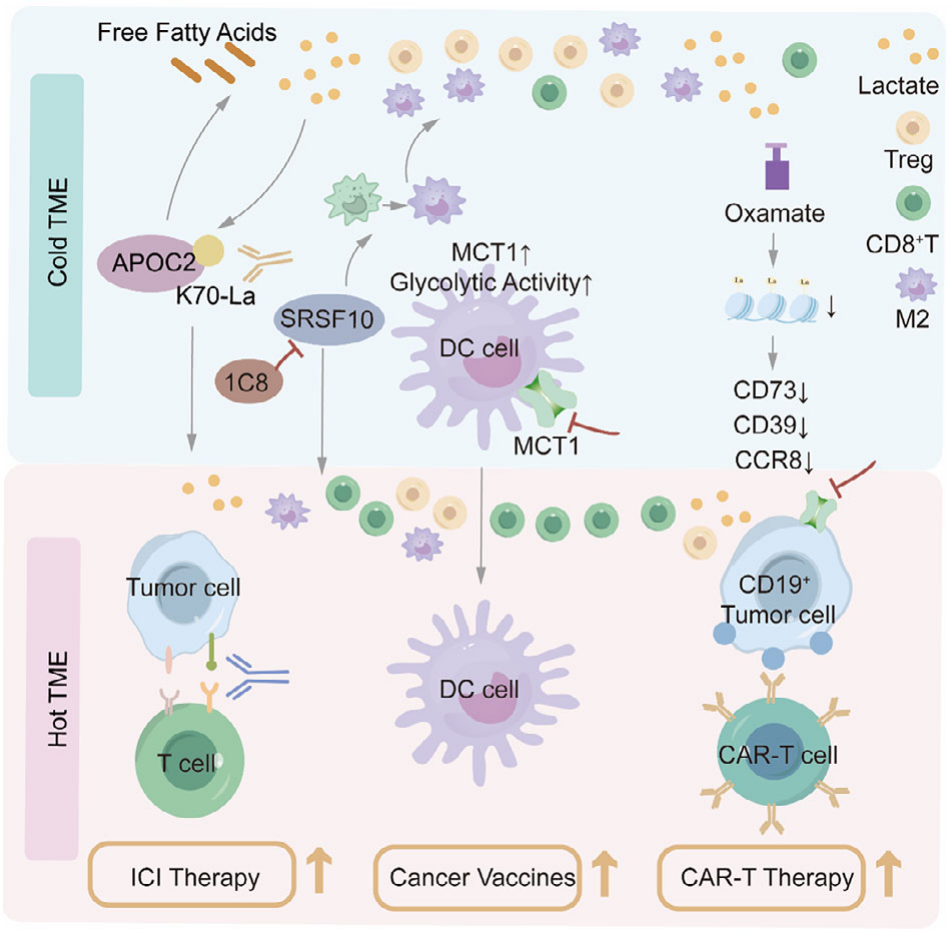
**Targeting lactate metabolism and lactylation enhances immunotherapy**. Schematic illustration showing how the inhibition of lactate production and histone lactylation reprograms the immunosuppressive TME. These interventions improve anti‐tumor immune responses and may synergize with ICI therapies, tumor vaccines, and CAR‐T therapies. Abbreviations: APOC2, Apolipoprotein C2; CAR‐T, Chimeric antigen receptor T cell; CCR8, C‐C chemokine receptor type 8; CD19, Cluster of differentiation 19; CD39, Cluster of differentiation 39; CD73, Cluster of differentiation 73; CD8^+^ T, Cytotoxic T lymphocyte; DC, Dendritic cell; ICI, Immune checkpoint inhibitor; K70‐La, Lysine‐70 lactylation; M2, M2‐polarized macrophage; MCT1, Monocarboxylate transporter 1; SRSF10, Serine/arginine‐rich splicing factor 10; TME, Tumor microenvironment; Treg, Regulatory T cell; 1C8, Small‐molecule inhibitor of SRSF10.

Targeting lactate metabolism and lactylation offers a promising avenue for enhancing cancer immunotherapy. By reshaping the immunosuppressive TME, these strategies can improve the efficacy of immune checkpoint inhibitors (ICIs), tumor vaccines, and chimeric antigen receptor (CAR)‐T cell therapies (Figure 6).

#### 4.4.1 Immune Checkpoint Inhibitors

The introduction of ICIs marks a milestone in cancer treatment; however, therapeutic resistance remains a major barrier to achieving durable clinical benefits. Several immune checkpoint receptors, including CTLA‐4 and PD‐1, are highly expressed on Tregs [253]. However, the role of these receptors on Tregs and their effects on ICI outcomes remains unclear.

Evidence indicates that PD‐1 expressed on Tregs may act as a negative regulator of their immunosuppressive ability. Pharmacologic blockade or genetic ablation of PD‐1 in vitro enhances the inhibitory ability of Tregs [254, 255]. In melanoma, anti‐PD‐1 treatment downregulates FOXP3 expression in peripheral blood monocytes, thereby reducing Treg function [256]. Metabolic regulation further modulates this interplay. Myc overexpression enhances PD‐1 expression by promoting glycolysis and creating a lactate‐rich TME, paradoxically increasing Treg‐mediated immunosuppression after PD‐1 blockade and resulting in therapeutic resistance. In preclinical models, inhibiting lactate metabolism in Tregs reverses such resistance [73]. Since lactate sustains the stability and function of Tregs, researchers found that combination therapy using an anti‐PD‐1 monoclonal antibody with an LDH inhibitor yields superior outcomes than anti‐PD‐1 alone in suppressing tumor progression [154].

Indeed, targeting lactylation enhances ICI efficacy. Lactate promotes lactylation of APOC2 at lysine 70 (K70), stabilizing the protein and facilitating free fatty acid release, Treg accumulation, immunotherapy resistance, and metastasis [123]. An antibody targeting APOC2‐K70 lactylation sensitized tumors to anti‐PD‐1 treatment [123]. Similarly, N‐Myc downstream regulated gene 1 (NDRG1) promotes lactate accumulation by stabilizing LDHA, leading to PD‐L1 upregulation, M2 macrophage recruitment, and reduced CTL infiltration. Dual targeting of NDRG1 and PD‐L1 yields synergistic anti‐tumor effects compared with either approach alone in murine lung adenocarcinoma models [257].

Although targeting lactate metabolism and lactylation shows promising synergy with ICIs, potential toxicity and immune‐related adverse events (irAEs) remain critical concerns. The combination of these strategies raises the possibility of compounded or novel toxicities. ICIs alone are known to induce irAEs through off‐target immune activation, affecting organs such as the gut, skin, liver, and lungs [258, 259, 260]. Because lactate metabolism is essential for normal physiology, its systemic disruption could exacerbate existing irAEs or introduce new forms of metabolic toxicity. However, to date, systematic studies evaluating these safety risks remain lacking.

#### 4.4.2 Cancer Vaccines

Cancer vaccines aim to elicit durable anti‐tumor immunity by targeting tumor‐specific or tumor‐associated antigens. However, complex immune‐evasion mechanisms and insufficient immunogenicity remain major challenges to their clinical efficacy [261]. Certain vaccines provide broader protection because they can trigger long‐lasting changes in innate immune memory. For example, following Bacillus Calmette‐Guérin (BCG) vaccination, increased lactate production correlates with increased cytokine responsiveness upon restimulation [262]. This effect is mediated by sustained H3K18 histone lactylation in monocytes, which endows long‐term innate immune memory and links epigenetic remodeling to amplified secondary inflammatory responses [262].

Metabolic reprogramming is also a key determinant of vaccine efficacy. Aerobic glycolysis is a defining metabolic feature of NK cell activation, and genetic ablation of LDHA impairs NK cell‐mediated anti‐viral defense and tumor surveillance [263]. In a phase I autologous vaccine clinical trial in patients with advanced melanoma, metabolic profiling of DCs revealed MCT1 upregulation and increased glycolytic activity. These alterations in DCs and circulating monocytes compromised vaccine efficacy by impairing endogenous antigen cross‐presentation [264].

Interestingly, high lactate concentrations can inhibit cancer cell proliferation and induce apoptosis in certain contexts [265]. In an in vivo study, tumor cells pretreated with lactate followed by irradiation functioned as an effective cancer vaccine, eliciting robust anti‐tumor immunity. [265] This approach increased DC phagocytic activity and promoted their maturation and aggregation [265]. Collectively, these findings suggest that targeting lactate metabolism represents a promising strategy to enhance vaccine‐induced immunity and the efficacy of cancer vaccines.

#### 4.4.3 Chimeric Antigen Receptor T Cell Therapy

CAR‐T therapy has achieved remarkable clinical success in hematologic malignancies but remains less effective against solid tumors due to the immunosuppressive TME and other inhibitory factors. In patients with diffuse large B‐cell lymphoma, the pre‐lymphodepletion ratio of lymphocytes to monocytes relative to serum LDH levels correlates with clinical outcome [266].

Pharmacologic modulation of lactate metabolism can potentiate CAR‐T cell function. In glioblastoma, the LDHA inhibitor oxamate enhances intratumoral CAR‐T cell activity by reducing histone H3K18 lactylation, which downregulates the promoter activity of CD39, CD73, and CCR8 [151]. This combination strategy of lactate inhibition plus CAR‐T significantly improved therapeutic efficacy [151]. Lactic acidosis rapidly suppresses CTL function, an effect reversed by pH normalization. Neutralizing extracellular acidity can restore CTL function, even in the presence of high lactate concentrations, offering a complementary means to enhance adoptive T cell therapy in solid tumors [267]. Exploiting metabolic differences between tumor cells and CAR‐T cells also provides a selective therapeutic window. In MCT1‐dependent lymphoma cells, but not in MCT1‐independent CAR‐T cells, inhibition of lactate transport selectively impairs tumor metabolism while enhancing CAR‐T cell anti‐tumor activity [210].

## 5 Concluding Remarks and Future Perspectives

Since its discovery, understanding of the Warburg effect in cancer has evolved considerably. Lactate, a central metabolite for metabolic reprogramming, shuttles between cells within the TME to sustain biosynthetic and energetic demands. Beyond its metabolic role, lactate functions as a signaling molecule that drives histone and non‐histone lactylation, a PTM that promotes cancer cell proliferation, metastasis, drug resistance, and immune tolerance. By linking metabolic reprogramming with epigenetic regulation, lactate and lactylation establish a positive feedback loop that provides new mechanistic insights into cancer progression. Nevertheless, essential questions persist regarding the compartments, kinetics, and site‐specific activities of lactylation, as well as how different cell populations within the TME integrate lactate signals to produce distinct phenotypic outcomes.

Promising preclinical advances have been made in targeting lactate production, transport, depletion, and/or buffering within the TME. Inhibition of lactate metabolism enhances the efficacy of both chemotherapy and immunotherapy [268, 269, 270, 271], and combination approaches yield synergistic outcomes [272, 273]. Additional progress includes lactate deprivation in cancer “starvation” therapy [274], and photodynamic and/or chemodynamic therapies that exploit lactate oxidation [275, 276]. Looking ahead, integrating lactylation sensing with metabolic and epigenetic interventions represents a compelling direction for precision cancer therapy. Such strategies could recondition the TME, restore anti‐tumor immunity, and potentially overcome resistance to cancer immunotherapy, represented by PD‐1/PD‐L1 blockade.

Despite these advances, major challenges remain to be addressed to translate lactate and lactylation research into clinical practice. First and foremost, in vivo evaluation of lactate metabolism in cancer remains elusive, especially direct measurements of elevated lactate in tumor interstitial fluid. This unmet need could be partially resolved by developing ultrasensitive, high‐resolution lactate sensors, a prerequisite for mapping the spatiotemporal dynamics of lactate metabolism and enabling clinical assessment [277]. Second, the threshold concentration of lactate required to effectively induce lactylation remains unknown. We are currently unable to distinguish the biological outcomes of individual lysine‐lactylation sites across different proteins. Defining minimum effective concentration and site‐specific functions will be essential for understanding how lactylation shapes cellular phenotypes in distinct tumor contexts. Third, the specificity of lactylation inhibitors remains inadequate for clinical application. Future efforts should prioritize high‐throughput screening for selective inhibitors and the development of highly sensitive, specific antibodies to detect these modifications in patient tumors. Such advances will also be crucial for biomarker development, mechanistic elucidation, and therapeutic strategies. Last but not least, the enzymatic machinery underlying the lactylation process remains incompletely characterized. The precise contribution of each lactylation enzyme is still in its infancy, particularly the specific “reader” proteins of lactylation. To clarify the enzymatic hierarchy of lactylation, comprehensive biochemical and structural analyses are needed to define substrate selectivity, catalytic efficiency, and regulatory dominance under varying metabolic conditions.

Moreover, lactate dysregulation is not unique to cancer. Metabolic diseases such as obesity and diabetes exhibit chronically elevated lactate production and systemic metabolic imbalance, owing to sustained glycolytic activation, adipose tissue hypoxia, and impaired mitochondrial function [168, 278, 279]. These conditions reshape innate and adaptive immune responses, promote low‐grade inflammation, and compromise immune‐cell function, changes that mechanistically parallel lactylation‐dependent pathways in tumors [278, 280]. Such systemic metabolic perturbations may amplify tumor lactate signaling, reinforce immune suppression, and regulate gene expression in both cancer and immune cells. Understanding how organism‐level metabolic dysfunction contributes to tumor lactate and lactylation biology may offer new opportunities to modulate anti‐cancer immunity and identify high‐risk patient groups with distinct metabolic and epigenetic liabilities.

Ultimately, elucidating how cancers exploit metabolic‒epigenetic circuits to evade immune surveillance will enable the design of innovative and durable cancer therapies. Rather than viewing lactate and lactylation solely as immunosuppressive barriers, future research should leverage their biological plasticity to transform metabolic vulnerabilities into therapeutic opportunities. A deeper mechanistic understanding of lactylation sensing, enzyme specificity, and metabolic regulation will be essential for developing next‐generation precision cancer therapies that integrate metabolism, epigenetics, and immuno‐oncology.

## Author Contributions

J.G. and Y.X. contributed equally to this work. J.G. and B.L. drafted the manuscript. L.L., J.G., B.L., Y.X., Z.W., and Y.W. edited the manuscript. J.G., Y.X., Z.W., and Y.W. prepared the figures. B.L. and L.L. critically reviewed the manuscript. All authors approved the final version of the manuscript.

## Conflicts of Interest

The authors declare that they have no conflicts of interest.
